# Coming Out to Doctors, Coming Out to “Everyone”: Understanding the Average Sequence of Transgender Identity Disclosures Using Social Media Data

**DOI:** 10.1089/trgh.2019.0045

**Published:** 2020-09-02

**Authors:** Oliver L. Haimson, Tiffany C. Veinot

**Affiliations:** School of Information, University of Michigan, Ann Arbor, Michigan, USA.

**Keywords:** transgender people, nonbinary people, transgender identity disclosure, health disparities, social media

## Abstract

**Purpose:** Gender transition is a complex life change, and transgender identity disclosures are pivotal moments that delineate the gender transition process. The purpose of this study was to quantify the average sequence in which transgender people disclose their transgender identity to different people in their lives, such as medical professionals, family members, and online networks, and to understand the emotional implications of these disclosures.

**Methods:** We used mixed methods to identify 362 transgender identity disclosure social media posts within 41,066 total posts from 240 Tumblr transition blogs (online spaces in which transgender people document gender transitions). We manually assigned each disclosure post an audience category, and then calculated the average sequence in which people in this sample disclosed their transgender identity to different audiences.

**Results:** Health professionals, such as physicians and therapists, were on average some of the very first people to whom transgender Tumblr bloggers disclosed their transgender identity. Such disclosures were often anxiety provoking and emotionally difficult, whether intentional or involuntary. Next, they often disclosed to friends, followed by close family (e.g., parents and siblings) and then extended family (e.g., grandparents). Mass disclosures to large portions of a person's network, such as on one's Facebook profile, usually came late in the disclosure process.

**Conclusion:** Gender transition is a staged process that includes a series of disclosures to different audiences that follows an average sequence. Because health care providers (e.g., physicians and therapists) who work with transgender patients are often some of the very first people to whom transgender people in our sample disclosed, providers must practice extra sensitivity when responding to such disclosures.

## Introduction

The dominant narrative of gender transition imagines an unambiguous, specific moment in which one's gender switches.^1^ However, transition is a process rather than a moment; it involves transgender identity disclosures to different people in one's life, some marking pivotal moments of change.^2^

When developing transgender identities, people balance their desires for a lived gender that matches their internal gender with considerations of available resources, coping abilities, and potential consequences of transition.^3^ Models of transgender identity transition have proposed five steps of coming out^4^ and 14 stages of identity development.^5^ However, transition can also be framed as a series of “milestones” reflecting diversity in transition goals,^6^ some of which involve disclosures. Although previous works offer important details regarding the temporal dynamics of transgender identity disclosure,^7^ we lack thorough empirical understanding of the average sequence of audiences to whom transgender people disclose. Knowledge of this sequence may help health care providers contextualize these disclosures, and react to them appropriately.

Emotional wellbeing shifts throughout gender transition processes, often temporally related to specific disclosures^8^—thus, uncovering the sequence in which transgender identity disclosures are likely to occur can illuminate emotional wellbeing patterns. Previous work with social media data showed that transgender identity disclosures included increased negative sentiment in the short term after disclosures to family members, increased positive sentiment after disclosures on Facebook, and overall increased positive sentiment.^8^ Transgender identity disclosures to family members impact participants' emotional wellbeing,^9^ and these emotions may change over time as family members become more accepting.^6^ Transgender experiences disclosing to friends are significant because friends play a major role in many transgender people's lives,^10^ and often become a “chosen family.” Disclosing one's transgender identity to a wide personal network on Facebook can be stressful, but support from one's network can mitigate distress.^11^ Understanding how each disclosure fits within the gender transition process can contextualize previous findings regarding emotions. We use a novel data source—social media—to understand the average sequence of transgender identity disclosures, a task that would be difficult using surveys or interviews given people's difficulty accurately recalling life events from the past.^12,13^

Disclosures to health care providers can have significant emotional implications. Many barriers exist for transgender people who need to disclose their transgender identity to receive medical care and counseling, such as fear of discrimination, stigma, privacy concerns, costs, and anxiety.^14–18^ Hence, health care providers play an important role in establishing a welcoming environment in which transgender patients feel comfortable disclosing.^19^ Nevertheless, health care professionals may lack clarity on how to best support transgender patients. Transgender health care may thus be improved with greater understanding of health care providers' typical place in transgender identity disclosure processes, and emotional responses to these disclosures. Therefore, we sought to understand the sequence in which transgender people in our sample disclosed their transgender identity to different audiences, where health care professionals fall within this sequence, and the emotional implications of transgender identity disclosures to health care professionals.

## Methods

Aspects of this study are drawn from a larger study; see^20^ for a more complete methods description. UC Irvine's Institutional Review Board (IRB) approved all study activities (HS#2014-1088).

### Data collection

**FIG. 1.**
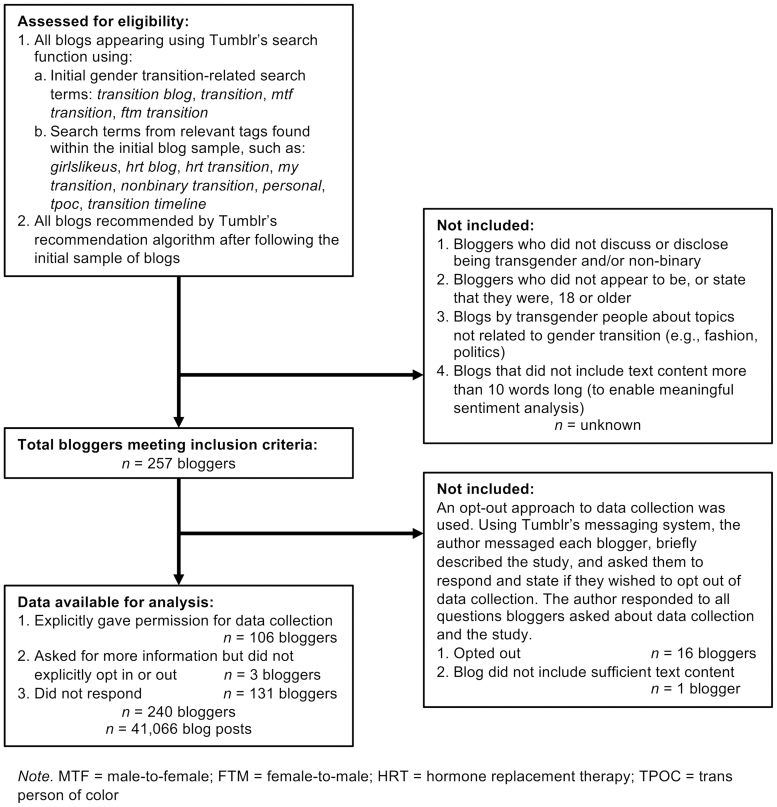
Data collection and inclusion criteria details for Tumblr transition blogs.

On the social media website Tumblr (www.tumblr.com), transition “blogs” are a common genre in which people document their gender transitions. These blogs include diary-like entries discussing social, medical, and legal aspects of transition: discussion of the coming out process and resulting acceptance, support, and/or rejection, physical and mental changes, medical procedures, and name and document changes. We collected text data from 240 transition blogs using the Tumblr API (application programming interface)^21^ and the PyTumblr API client,^22^ an approach aligned with Tumblr's API License Agreement as of January 2017.^23^ Figure 1 displays our blog inclusion criteria and data collection approach.

### Determining transgender identity disclosures in the dataset

We define Tumblr transgender identity disclosure posts as those describing transgender identity disclosures in the past two weeks—recent enough to be relatively accurate, which is necessary for our longitudinal approach. To locate these posts in the dataset, two researchers coded 50 posts as either transgender identity disclosures or not (to establish interrater reliability), and one then coded the remaining 1,150 training data posts. Then, to identify a larger set of posts, we built a machine learning classifier, which is a computational method of predicting categories in a dataset given an annotated training dataset. We chose this computational method because the dataset was too large to code manually (41,066 total posts).

After testing nine machine learning algorithms, we found that the AdaBoost algorithm^24^ was most accurate: when applying 10-fold cross validation, it had accuracy of 0.80 and area under the curve (AUC) of 0.62. To further test a classifier, it must be applied to data it has not seen before.^25^ When applying the classifier to the 20% of data held out as a test set, its accuracy was 0.79 and AUC was 0.71. Further details of this method are detailed in previous publications.^8,20^ To identify disclosure posts, we then applied the classifier to the full dataset. The model classified 798 posts as discussing transgender identity disclosures. We then manually coded these posts to ensure that all were indeed focused on disclosure. Through manual coding, we identified 362 total posts describing recent transgender identity disclosures.

### Data analyses

Using qualitative open coding^26^ followed by directed coding,^27^ we manually identified the disclosure audience(s) for each transgender identity disclosure post by reading the post. Next, we quantified the average sequence of transgender identity disclosure audiences. For each person who had posted about more than one transgender identity disclosure on their blog, we assigned a weight to each disclosure audience as follows: for each person who had posted about disclosing to that audience, we divided the sequence of that disclosure by the total number of transgender identity disclosures from that person. For example, if a person had made three total disclosures: first they disclosed to their mother, second at school, and third on Facebook, then for that person the maternal disclosure would receive a weight of 1/3, the school disclosure a weight of 2/3, and the Facebook disclosure a weight of 1. Then we averaged the weight each audience received over all people who had posted about that disclosure audience. Thus, each audience received a weight between zero and one, where a lower number indicates a disclosure earlier in the transition process, and a higher number indicates a disclosure later in the process.

Finally, we qualitatively analyzed posts that involved disclosures to health care providers, using an inductive open-coding process in which we allowed codes and themes to arise from the data.^26^ This enabled us to understand the types of health professionals to whom people disclosed, and reasons for these disclosures. An emergent theme involved emotional implications of disclosure to health care providers, which is discussed in the Results section.

All bloggers were contacted and given the opportunity to opt out of data collection (Fig. 1). All quotes included in this study are either nontraceable through Google search, or paraphrased.

## Results

### Characteristics of bloggers and blogs

We obtained demographic data (Table 1) from blog descriptions and posts. Table 2 provides blog characteristics. The dataset ranged from 2009 to 2017.

**Table 1.** Blogger Demographics

Demographic	n	Percentage
Gender^a^		
Transgender man	113	47
Transgender woman	110	46
Nonbinary person	17	7
Age,^b^ years		
18–24	147	63
25–34	68	30
35–44	13	7
45 or over	1	<1

^a^ Some bloggers identified as more than one gender (e.g., transgender man and nonbinary), yet are categorized here as the most prominent gender category described on their blog.

^b^ Ages skew young, as Tumblr does more broadly.

Race/ethnicity data were not available, as 93% of bloggers did not specify this on their blogs.

**Table 2.** Blog Characteristics

Blog characteristic	Mean	Median	Standard deviation
Post word count	71.38	33	124.70
Posts per blogger	367.31	76	814.46
Number of days blog active	645.57	530	515.19
Number of transgender identity disclosures per blogger	1.51	0	2.98
Number of transgender identity disclosures per blogger for those with more than one disclosure	5.25	4	3.97
Post year (data collected in January 2017)	2017 (0.3%); 2016 (35.2%); 2015 (30.5%); 2014 (17.7%); 2013 (10.1%); 2012 (4.1%); 2011 or earlier (2.1%)		

### Average disclosure sequence

**FIG. 2.**
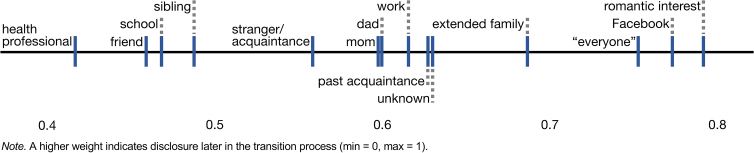
Relative transgender identity disclosure audience sequence on average.

We identified 20 primary disclosure audience types (Table 3). Table 3 and Figure 2 show the average sequence in which transition bloggers disclosed their transgender identity to people in their lives. Disclosures to self and to one's spouse/romantic partner do not appear because for most these disclosures happened before they started their transition blog.

**Table 3.** Disclosure Audience Sequence on Average

Audience	Mean/weight	Standard deviation	n
Health professional	0.417	0.225	11
Friend	0.459	0.257	57
School	0.468	0.238	18
Sibling	0.488	0.278	29
Stranger/acquaintance	0.559	0.300	65
Mother	0.599	0.273	35
Father	0.599	0.298	28
Work	0.616	0.273	77
Past acquaintance	0.628	0.372	10
Unknown	0.629	0.290	15
Extended family	0.687^*^	0.311	56
Everyone	0.753	0.313	13
Facebook	0.773^**^	0.221	26
Romantic interest	0.792^*^	0.182	8

Category significantly different from health professional category, based on two-sample *t*-tests with Bonferroni adjusted significance levels (α/13): ^*^p<0.0038; ^**^p<0.0008.

Categories with too few instances to calculate meaningful statistics: partner, ex-partner, child, church, Instagram, Tumblr, Twitter. A higher mean indicates disclosure later in the transition process.

For those who disclosed to health professionals, these types of disclosures typically happened early in the process. Disclosures to friends also happened early, family members toward the middle of the process, and more broad disclosures as a later step. Facebook disclosures happened in close proximity to disclosures to “everyone,” indicating that many Tumblr transition bloggers disclosed on Facebook when ready to inform their broader network.^2^

As stated, disclosures to health professionals are, on average, early; in fact, other than self and romantic partners, health professionals are often the very first people to whom bloggers disclosed. These early disclosures were typically to therapists or counselors; medical professionals such as physicians; or other transition-related health care providers like laser hair removal technicians. Therapists/counselors could be resources for discussing future disclosures, and for accessing letters required for other providers. Physicians included doctors whom a person sees to discuss steps to starting on hormone replacement therapy (e.g., endocrinologists) and primary care providers who may provide advice on medical transition.

Disclosures to romantic interests tended to happen after others, even after Facebook disclosures. This is because new romantic interests often did not know the person before transition and may not be in their Facebook network—and because disclosure to a romantic interest may be necessary even after transition. Disclosures to acquaintances and strangers took place throughout gender transition and beyond. Bloggers often explained these as unplanned disclosures that happened as they ran into such people throughout the course of their transitions and lives.

**FIG. 3.**
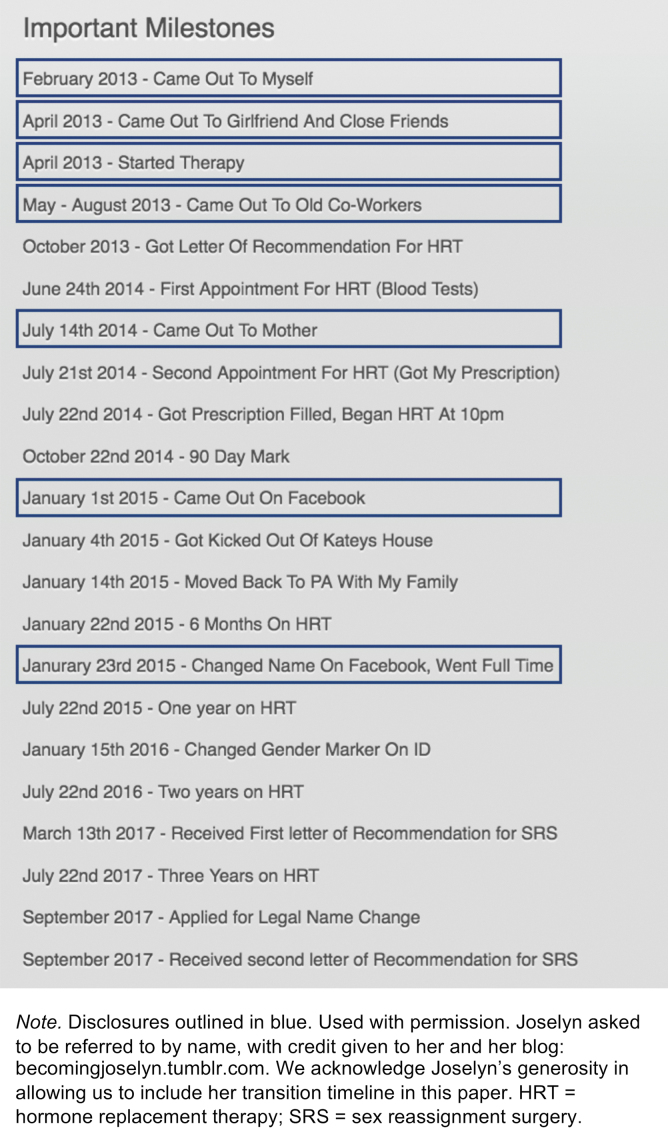
Joselyn's important milestones.

Despite this average sequence that we present, transgender identity disclosures can come in any order depending upon personal circumstances. To illustrate, Figure 3 shows Joselyn's (from the blog Becoming Joselyn) “Important Milestones.” Joselyn follows the general trend of coming out to friends and a therapist early in the process, then family, and then finally Facebook. In her case, the Facebook disclosure took two steps—an announcement, followed by a later name change. Yet, in some ways Joselyn's journey differs from the average. She came out to her close friends before disclosing to a health professional. She also disclosed to coworkers sooner than most. Joselyn's milestones list also highlights that disclosures are interspersed within other pivotal transition moments, such as beginning hormones, planning for surgery, and steps toward legal name change.

### Emotional implications of disclosure to health care providers

We report on two qualitative themes—(1) intentional and (2) involuntary transgender identity disclosures to providers—and their emotional implications, including anxiety and distress.

Some early disclosures to health care providers were intentional and deliberately sought; nevertheless, they were often anxiety provoking and emotionally draining. Bloggers' posts reflected the emotional demands of finding transgender-competent health care providers to whom disclosure would be emotionally safe. For instance, in seeking transgender-competent care, one blogger stated: *“After hours of phone calls, suggestions, coming out to strangers (and even my endocrinologist) every few minutes yesterday, I still had no luck finding a trans friendly doctor who prescribes hormones.”* Once a provider has been selected, bloggers also expressed fear concerning initial disclosures to them, both verbal and physical appearance related. One blogger described an encounter with her therapist: *“It's the first time I've worn a dress in front of anyone…She told me that she was proud of me, and asked…how I was feeling. Nervous, of course. It took a few minutes sitting down talking with her to get my mind off the fact that I had a dress on and we were chatting.”* This post exemplifies the anxiety salient for many in transgender identity disclosures to health care providers.

Transgender identity disclosures to health care professionals could also be involuntary, especially if emerging from medical necessities not linked to gender transition. Such unwanted disclosures are likely to happen early in transition, when people's medical records may not match their current name or appearance. For example, one blogger described an experience at a dentist's office that involved her phoning a previous (nontransition-related) surgeon to answer a question: *“Before I knew it, I was having to tell them my old name so that the surgeon's office could look up my records.”* This event involved multiple disclosures as she had to disclose her transgender status to the surgeon's office staff through phone, the dentist's office staff, and the dentist—all in one office visit. For this blogger, although *“it had the potential to be the worst ever,”* the experience was affirming because providers treated her *“like a decent, normal human being.”*

## Discussion

We analyzed 240 Tumblr transition blogs to create a timeline of relative audience disclosure sequence on average. Health professionals, such as therapists and physicians, were often some of the very first people to whom transgender people in our sample disclosed their transgender identity. Friends came next, followed by close family and extended family. Mass disclosures, such as on Facebook, usually came late in the process. Bloggers described both voluntary and involuntary disclosures, and anxiety associated with both types.

Given the prominence of before-and-after visuals and narratives in mainstream and social media, many assume that gender transition is a relatively simple and quick event.^28^ Quantifying the average transgender identity disclosure sequence provides evidence that gender transition is instead a *process* that happens over time. Findings confirm previous research showing that people disclosed to friends early in the disclosure process, followed by siblings and mothers.^7^ Yet, longitudinal social media data enabled us to analyze a sample of people's disclosure experiences, written in their own words at the time of the disclosure. This type of data is potentially more accurate than previous work that has captured similar information using retrospective reporting, which is subject to recall bias.^13^ These methods enabled us to analyze people's rich, real-time descriptions of their emotions around transgender identity disclosures, such as the anxiety that often accompanied disclosures to health care professionals.

Health care provider sensitivity is vital given that, for those transgender people who desire medical transition, barriers to medical transition hinder transition's positive effects^29,30^ and can contribute to poor health.^15^ In addition, many transgender people delay health care because they fear discrimination, which poses further health challenges.^31^ Compounded with the distress we documented involving early transgender identity disclosures, this can make for particularly difficult office visits. When a person discloses their transgender identity, physicians and therapists may not realize that they are likely to be one of the first audiences to whom that person has disclosed—possibly second only to partners/spouses. Thus, responding to this disclosure requires a level of sensitivity that health care providers may not currently feel prepared to provide, particularly if they assume that the patient is used to disclosing and fielding responses from others.

Considering how early in the transition process disclosures to health care providers are, a positive, affirming, supportive response to a patient's transgender identity disclosure is paramount. Although health care provider sensitivity and respect toward transgender people is important regardless of disclosure sequence, early disclosures provide an opportunity for providers to *establish* a supportive health care environment. Doing so successfully may encourage a transgender person to continue to seek out care. Providers can improve their responses to transgender identity disclosures by educating themselves on transgender experiences and health needs,^32–35^ learning how to advocate for patients, and changing policies to be transgender inclusive.^36^ Therapists and counselors can learn by reading the American Psychological Association's “Guidelines for Psychological Practice With Transgender and Gender Nonconforming People,”^34^ which recommends acknowledging (to name a few): gender's complexity; the diversity of transgender people's experiences; health care disparities, barriers, and stigma facing transgender people; and the necessity of transgender-affirmative health care. Physicians and therapists unfamiliar with transgender patients and their needs should consider hiring on-call patient advocates to provide support to transgender patients during these early transition office visits, which would help ease some of the emotional distress we found that people experienced around provider visits.

The disclosure sequence timeline could help therapists understand what to expect during gender transition and how to best support transgender patients. Without substantial experience working with transgender clients, a therapist would be unlikely to know what types of disclosures would commonly come next for their client. Although the exact sequence will be different for each person, having an overall average “roadmap” would be helpful for providers to best support their clients throughout the transition process. This suggestion is in line with previous work advocating that counselors can help transgender people with planning their transition and providing support around difficult disclosures,^34,37^ yet adds a new tool to assist.

Finally, the fact that transgender people often disclose to health professionals so early distinguishes transgender identity disclosure from lesbian, gay, bisexual, and queer (LGBQ) sexual identity disclosures, which usually do not begin with disclosures to health professionals.^38^ Unlike LGBQ identity processes, gender transition (if one chooses to transition medically) is highly medicalized and often involves substantial gatekeeping. That is, to begin medical transition, a transgender person must involve a physician or nurse practitioner, and accessing these providers often requires a referral from a therapist^39^ (although many clinics are moving toward more progressive models such as informed consent). In contrast, coming out as LGBQ does not require medical interventions or a “doctor's note” to affirm one's identity. Health care providers should be prepared to support patients within the highly medicalized gender transition process, and understand that transgender patients may be visiting them out of necessity to surmount certain barriers involved in gatekeeping. Still, providers should understand that not all transgender people pursue or desire medical transition, and be attuned to the patient's stated purpose of the visit (which may not be related to their transition) rather than making assumptions.

This study involves several limitations. First, results relied upon data from transgender Tumblr users, who may differ from the broader population. Our sample does not include minors or older adults, and disclosure sequences may be different for these populations. Tumblr posts may not be complete or accurate representations of bloggers' experiences and emotions; posts may include more emotionally significant events. Only some bloggers posted about identity disclosures, and there were a small number of posts about health care providers, which leads to overweighting of specific individuals in the results. In addition, our dataset includes very little data about disclosures to spouses/partners. Next, our machine learning model to detect transgender identity disclosures may not have detected all relevant disclosure posts. Finally, although disclosures are important transition milestones for many people,^2,6^ they should not be considered requirements or measures of transition success.

## Conclusion

Health care providers are some of the first audiences to whom those in our sample disclosed their transgender identity. Disclosures to friends came next, followed by disclosures to family members. Mass disclosures to one's Facebook network (“everyone”) happened late in the disclosure process. These results provide evidence that gender transition consists of different types of disclosures to different audiences, rather than a single event. Bloggers described distress surrounding voluntary and involuntary disclosures to health care providers. Health care providers should practice extra sensitivity in these situations, and should be supported in accessing resources and developing skills to enhance their sensitivity. Transgender people may also benefit from supplemental support to ease the anxiety of disclosures to health care providers.

## Abbreviations Used

AUC: area under the curve
IRB: Institutional Review Board
LGBQ: lesbian, gay, bisexual, and queer

## References

[1] Stone AR (Sandy). The Empire Strikes Back: A Posttranssexual Manifesto. 1987. Available from: http://sandystone.com/empire-strikes-back.pdf Accessed 716, 2014

[2] Haimson OL. Social media as social transition machinery. Proc ACM Hum-Comput Interact. 2018;2(CSCW):63:1–27

[3] Levitt HM, Ippolito MR. Being transgender: the experience of transgender identity development. J Homosex. 2014;61:1727–1758 25089681 10.1080/00918369.2014.951262

[4] Bockting W, Coleman E. Developmental stages of the transgender coming-out process: toward an integrated identity. In: Principles of Transgender Medicine and Surgery. (Ettner R, Monstrey S, Coleman E; eds). Abingdon-on-Thames, UK: Routledge/Taylor & Francis Group, 2016, pp. 137–158

[5] Devor AH. Witnessing and mirroring: a fourteen stage model of transsexual identity formation. J Gay Lesbian Psychother. 2004;8:41–67

[6] Beemyn G, Rankin S. The Lives of Transgender People. New York: Columbia University Press, 2011

[7] Maguen S, Shipherd JC, Harris HN, Welch LP. Prevalence and predictors of disclosure of transgender identity. Int J Sex Health. 2007;19:3–13

[8] Haimson OL. Mapping gender transition sentiment patterns via social media data: toward decreasing transgender mental health disparities. J Am Med Inform Assoc. 2019;26:749–758 31120498 10.1093/jamia/ocz056 PMC6696505

[9] Veldorale-Griffin A. Transgender parents and their adult children's experiences of disclosure and transition. J GLBT Fam Stud. 2014;10:475–501

[10] Galupo MP, Krum TE, Hagen DB, et al. Disclosure of transgender identity and status in the context of friendship. J LGBT Issues Couns. 2014;8:25–42

[11] Haimson OL, Brubaker JR, Dombrowski L, Hayes GR. Disclosure, Stress, and Support During Gender Transition on Facebook. Proc 18th ACM Conf Comput Support Coop Work Soc Comput. New York, NY: ACM, 2015, pp. 1176–1190. Available from: http://doi.acm.org/10.1145/2675133.2675152 Accessed 522, 2015

[12] Mitchell TR, Thompson L, Peterson E, Cronk R. Temporal adjustments in the evaluation of events: the “Rosy View.” J Exp Soc Psychol. 1997;33:421–448 9247371 10.1006/jesp.1997.1333

[13] Jenkins CD, Hurst MW, Rose RM. Life changes: do people really remember? Arch Gen Psychiatry. 1979;36:379–384 426603 10.1001/archpsyc.1979.01780040021001

[14] Alegria CA. Transgender identity and health care: implications for psychosocial and physical evaluation: transgender identity and health care. J Am Acad Nurse Pract. 2011;23:175–182 21489011 10.1111/j.1745-7599.2010.00595.x

[15] White Hughto JM, Reisner SL, Pachankis JE. Transgender stigma and health: a critical review of stigma determinants, mechanisms, and interventions. Soc Sci Med. 2015;147:222–231 26599625 10.1016/j.socscimed.2015.11.010 PMC4689648

[16] Shipherd JC, Green KE, Abramovitz S. Transgender clients: identifying and minimizing barriers to mental health treatment. J Gay Lesbian Ment Health. 2010;14:94–108

[17] Benson KE. Seeking support: transgender client experiences with mental health services. J Fem Fam Ther. 2013;25:17–40

[18] White Hughto JM, Rose AJ, Pachankis JE, Reisner SL. Barriers to gender transition-related healthcare: identifying underserved transgender adults in Massachusetts. Transgend Health. 2017;2:107–118 29082331 10.1089/trgh.2017.0014 PMC5627670

[19] Reisner SL, Bradford J, Hopwood R, et al. Comprehensive transgender healthcare: the gender affirming clinical and public health model of Fenway Health. J Urban Health. 2015;92:584–592 25779756 10.1007/s11524-015-9947-2 PMC4456472

[20] Haimson OL. The Social Complexities of Transgender Identity Disclosure on Social Media. Irvine: University of California, 2018. Available from: https://escholarship.org/uc/item/19c235q0 Accessed 81, 2018

[21] Tumblr. Tumblr API. Available from: https://www.tumblr.com/docs/en/api/v2 Accessed 1215, 2016

[22] PyTumblr. PyTumblr. GitHub. Available from: https://github.com/tumblr/pytumblr Accessed 1215, 2016

[23] Tumblr. Application Developer and AP I License Agreement | Tumblr. 2014. Available from: https://www.tumblr.com/docs/en/api_agreement Accessed 730, 2016

[24] sklearn.ensemble.AdaBoostClassifier—scikit-learn 0.21.2 documentation. Available from: https://scikit-learn.org/stable/modules/generated/sklearn.ensemble.AdaBoostClassifier.html Accessed 711, 2019

[25] Witten IH, Frank E, Hall MA. Data Mining: Practical Machine Learning Tools and Techniques. Burlington, MA: Elsevier, 2011

[26] Strauss A, Corbin JM. Basics of Qualitative Research: Techniques and Procedures for Developing Grounded Theory. Thousand Oaks, CA: SAGE Publications, 1998

[27] Hsieh H-F, Shannon SE. Three approaches to qualitative content analysis. Qual Health Res. 2005;15:1277–1288 16204405 10.1177/1049732305276687

[28] Prosser J. Second Skins: The Body Narratives of Transsexuality. Columbia: University Press, 1998

[29] What We Know: The Public Policy Research Portal, Center for the Study of Inequality at Cornell University. What does the scholarly research say about the effect of gender transition on transgender well-being? What We Know. 2019. Available from: https://whatweknow.inequality.cornell.edu/topics/lgbt-equality/what-does-the-scholarly-research-say-about-the-well-being-of-transgender-people/ Accessed 13, 2019

[30] Kozee HB, Tylka TL, Bauerband LA. Measuring transgender individuals' comfort with gender identity and appearance development and validation of the transgender congruence scale. Psychol Women Q. 2012;36:179–196

[31] Seelman KL, Colón-Diaz MJP, LeCroix RH, et al. Transgender noninclusive healthcare and delaying care because of fear: connections to general health and mental health among transgender adults. Transgend Health. 2017;2:17–28 28861545 10.1089/trgh.2016.0024 PMC5436369

[32] National Center for Transgender Equality. Issues: Health & HIV. National Center for Transgender Equality. Available from: https://transequality.org/issues/health-hiv Accessed 711, 2019

[33] Deutsch MB. Guidelines for the Primary and Gender-Affirming Care of Transgender and Gender Nonbinary People. UCSF Center of Excellence for Transgender Health. 2016. Available from: http://www.transhealth.ucsf.edu/trans?page=protocol-00-00 Accessed 711, 2019

[34] American Psychological Association. Guidelines for psychological practice with transgender and gender nonconforming people. Am Psychol. 2015;70:832–864 26653312 10.1037/a0039906

[35] Carlozzi A. Counseling transgender persons and their families. Counseling Today. 2017. Available from: https://ct.counseling.org/2017/08/counseling-transgender-persons-families/ Accessed 711, 2019

[36] Rossman K, Salamanca P, Macapagal K. A qualitative study examining young adults' experiences of disclosure and nondisclosure of LGBTQ identity to health care providers. J Homosex. 2017;64:1390–1410 28459379 10.1080/00918369.2017.1321379 PMC5772907

[37] Bockting WO, Knudson G, Goldberg JM. Counseling and mental health care for transgender adults and loved ones. Int J Transgend. 2006;9:35–82

[38] Corrigan P, Matthews A. Stigma and disclosure: implications for coming out of the closet. J Ment Health. 2003;12:235–248

[39] Denny D. Changing models of transsexualism. J Gay Lesbian Psychother. 2004;8:25–40

